# Bayesian inference for Cox proportional hazard models with partial likelihoods, nonlinear covariate effects and correlated observations

**DOI:** 10.1177/09622802221134172

**Published:** 2022-11-01

**Authors:** Ziang Zhang, Alex Stringer, Patrick Brown, Jamie Stafford

**Affiliations:** 1 Department of Statistical Science, 7938 University of Toronto, Toronto, Canada; 2 Department of Statistics and Actuarial Sciences, 8430 University of Waterloo, Waterloo, Canada; 3 Centre for Global Health Research, 10071 St Michael’s Hospital, Toronto, Canada

**Keywords:** Cox proportional hazard model, partial likelihood, approximate Bayesian inference, hierarchical modeling

## Abstract

We propose a flexible and scalable approximate Bayesian inference methodology for the Cox Proportional Hazards model with partial likelihood. The model we consider includes nonlinear covariate effects and correlated survival times. The proposed method is based on nested approximations and adaptive quadrature, and the computational burden of working with the log-partial likelihood is mitigated through automatic differentiation and Laplace approximation. We provide two simulation studies to show the accuracy of the proposed approach, compared with the existing methods. We demonstrate the practical utility of our method and its computational advantages over Markov Chain Monte Carlo methods through the analysis of Kidney infection times, which are paired, and the analysis of Leukemia survival times with a semi-parametric covariate effect and spatial variation.

## 1. Introduction

For problems involving time-to-event data, the combination of Cox proportional hazard (Cox PH) models and inference via partial likelihood has been the dominant methodology following its development by Cox.^1^ The Cox PH model assumes that any two subjects’ event hazards are proportional as a function of time, with the ratio depending on unknown covariate effects which are inferred from the observed data. Event times may be correlated within the sample, for example when the response is time to kidney failure for the left and right kidneys from the same subject. Inference that is conducted via partial likelihood does not require assumptions to be made about the form of the baseline hazard. Further, the use of Bayesian inference with the Cox PH model is desirable as this (a) allows the use of substantive prior information on the hazard ratios and (b) provides uncertainty quantification for all parameters of interest in the presence of complex models for the hazard, which would be difficult to achieve otherwise. However, existing methods for approximate Bayesian inference based on integrated nested Laplace approximations (INLA)^2^ cannot be applied to the Cox PH model with partial likelihood because the Hessian matrix of the log partial-likelihood is fully dense while INLA requires this matrix to be diagonal.

Alternative methods of making Bayesian inference for this kind of survival model have been considered in the literature. Dykstra and Laud^3^ considered a fully non-parametric approach for Bayesian survival analysis, where the entire hazard function is modeled with an extended gamma process prior and the posterior distribution is derived to be another extended gamma process. Kim and Kim^4^ considered Bayesian analysis on Cox PH model on partial likelihood and on full likelihood with an extended gamma process prior for the baseline hazard, and carried out inferences using Markov Chain Monte Carlo (MCMC). Martino et al.^5^ considered application of the INLA methodology to the Cox PH model, using the full likelihood with baseline hazard modeled semi-parametrically. Kalbfleisch^6^ derived the partial likelihood to be the limiting posterior when baseline hazards are modeled with non-informative priors, and Sinha et al.^7^ later extended the result to allow the inclusion of grouped survival data, and implemented partial likelihood-based Bayesian inference with a Gibbs sampling algorithm. Henschel et al.^8^ proposed a Bayesian inference method using MCMC on the full likelihood, with baseline hazard function modeled either as piecewise constant or as linear combination of B-splines, and they accommodated the inclusion of different types of frailties in their method. Hennerfeind et al.^9^ developed a general geo-additive Cox PH model that allows the inclusion of components such as non-linear covariate effect, spatial effect and group level frailties, with inference carried out using MCMC on full likelihood and baseline hazards modeled using P-splines. Kneib^10^ generalized the method of Hennerfeind et al.^9^ to accommodate left truncation, left censoring, and interval censoring of the survival times. Most of the existing methods for Bayesian inference of Cox PH model have been utilizing MCMC method to obtain the posterior, and are based on the full likelihood with an explicit form to model the unknown baseline hazard.

Stringer et al.^11^ developed an approximate Bayesian inference methodology for case-crossover model, which can be viewed as a special case of Cox PH model, by applying the approximation strategy of INLA to a log-partial likelihood with a non-diagonal Hessian matrix. Their methodology includes nonlinear covariate effects and yields full posterior uncertainty for the corresponding smoothness parameters, an improvement over existing frequentist methods. The partial likelihood they considered corresponds to one of the simplest special case[s] of the general Cox PH model, and the Hessian matrix of their log-partial likelihood is block-diagonal and sparse. In contrast, the Hessian matrix of log-partial likelihood of Cox PH model is generally fully dense, leading to increased computational burden when compared to the model considered by Stringer et al.^11^ Further, they use a manual integration strategy which requires the user to supply their own quadrature points, which requires specialist knowledge to do properly. This limits the practical utility of their method. In order to make approximate Bayesian inferences for the Cox PH model with partial likelihood, an alternative computational strategy is needed.

In this paper, we develop an approximate Bayesian inference method for Cox PH models with partial likelihood, that allows linear and nonlinear covariate effects, spatial effects and frailties for modeling correlations between survival times. The proposed inference method utilizes the Laplace approximation-based strategy^2,11^ in a novel way that accommodates the use of partial likelihood. Through two simulation studies, we illustrate the circumstances under which the proposed method yields improved results compared to existing methods based on full likelihood, and demonstrate the accuracy of the posterior approximation and its computational advantages compared to partial likelihood method fit with MCMC. We then further demonstrate the practical utility of the proposed method through the analysis of two classical datasets with correlated survival times, nonlinear covariate effects and spatial variations.

The remainder of this paper is organized as follows. In section 2 we describe the Cox PH model and the method of semi-parametric smoothing that will be used in the inference of the nonlinear covariate effect in this paper. In section 3, we describe our proposed methodology and the introduced improvements to solve the computational challenges presented by the complicated partial likelihood. In section 4 we illustrate advantages of the proposed methodology in two simulation studies and through the analysis of the Kidney catheter data analyzed by McGilchrist and Aisbett^12^ and Leukemia survival data analyzed by Martino et al.^5^ We conclude in section 5 with a discussion.

## 2. Model

### 2.1. A general Cox PH model

Suppose we observe n groups indexed by i, each with $n_{i}$ observations indexed by j. For example, we may observe n subjects with $n_{i}$ measurements per subject. Denote the random variable representing the jth survival time in the ith group by $T_{ij}$, and denote its realization by $t_{ij}$. Let $c_{ij}$ denote the censoring time for observation $T_{ij}$ such that $T_{ij}$ is not directly observable when $c_{ij}<T_{ij}$. The observed survival time is $y_{ij}=\min\{t_{ij},c_{ij}\}$. Define $d_{ij}=1$ if $y_{ij}=t_{ij}$ (a survival time) and $d_{ij}=0$ if $t_{ij}>y_{ij}$ (a censoring time). The observations for each i,j are hence denoted by pairs $y=\{(y_{ij},d_{ij})\;:\;i\in [n];j\in [n_{i}]\}$. The total number of rows in the data set is denoted by $N=\sum_{i=1}^{n}n_{i}$. By default, we assume the assumption of independent censoring holds to ensure valid inferences from the Cox PH model, which states that the hazard functions conditional on the same covariate values are the same between the censored and uncensored individuals.^13^

Define $h_{ij}(t)$ to be the hazard function for the random variable $T_{ij}$. The Cox PH model assumes $h_{ij}(t)=h_{0}(t)\text{exp}(\eta_{ij})$ where $h_{0}(t)$ is an unknown baseline hazard function that does not depend on the covariates. Kim and Kim^4^ considered the inference on linear fixed effects with linear predictor defined as $\eta_{ij}={x_{ij}}^{T}\beta$, and they briefly discussed the possibility of generalizing their method to accommodate survival times correlated within groups. Sinha et al.^7^ proposed a MCMC method for inference with both fixed effects and group level frailties using partial likelihood, but their method does not accommodate nonlinear covariate effect. Dykstra and Laud^3^ on the other hand considered to model the entire hazard function $h_{ij}(t)$ nonparametrically, but their method cannot be directly used to quantify the association of a particular covariate with the survival times.

Stringer et al.^11^ considered a general linear predictor that accommodates both linear fixed and nonlinear semi-parametric covariate effects, but the type of likelihood they considered is one of the simplest special cases of the general partial likelihood of Cox PH model, and does not allow the estimation of group-level correlation of survival times.

To accommodate nonlinear covariate effects and correlated survival times, we define an additive predictor $\eta_{ij}$ which links the covariates for the ijth observation to the survival time $T_{ij}$:

(1) $$\begin{array}{cc} \eta_{ij} & ={x_{ij}}^{T}\beta +\sum_{q=1}^{r}\gamma_{q}(u_{qij})+\xi_{i},i\in [n],j\in [n_{i}],\\ \xi_{i}|\sigma_{\xi } & \overset{iid}{∼}N(0,\sigma_{\xi }),i\in [n],\\ \gamma_{q}(\cdot )|\sigma_{q} & \overset{ind}{∼}GP\left(0,C_{\sigma_{q}}\right),q\in [r]. \end{array}$$

Let $\eta =\{\eta_{ij}\;:\;i\in [n];j\in [n_{i}]\}$ be the vector of all the additive linear predictors. Here $x_{ij}$ is a p-dimensional vector of covariates that are modeled as having linear associations with the log-hazard, and $\beta =(\beta_{1},\ldots ,\beta_{\;p})$ are regression coefficients. The $u_{q}=\{u_{qij}\;:\;i\in [n];j\in [n_{i}]\},q\in [r]$ are covariates whose associations with the log-hazard are modeled semi-parametrically through unknown smooth functions $\left\{\gamma_{i},i\in [r]\right\}$. The vector of group intercepts $\xi =\{\xi_{i}\;:\;i\in [n]\}$—referred to as *frailties* coefficients in the context of survival analysis^14^—are included to model correlation between survival times coming from the same group i. There is no global intercept $\beta_{0}$ as this would be absorbed by $h_{0}(t)$. However, in contrast to the case-crossover model considered by Stringer et al.,^11^ the group-specific intercepts $\xi_{i}$ are estimable in this general Cox PH model, since the case-crossover model only compares the survival times within the same group but the Cox PH model compares all the survival times across all groups.

### 2.2. Modeling nonlinear covariate effect

The nonlinear covariate effects $\gamma_{q}$, q∈[r] are modeled semi-parametrically as r∈N independent zero-mean Gaussian processes, each defined by its covariance function $C_{\sigma_{q}}$. The covariance functions are each parametrized by a single parameter $\sigma_{q}>0$. A typical choice of covariance function is the covariance function of the second fold Integrated Wiener process,^15^ which has a connection to cubic smoothing splines.^16^

To infer the infinite-dimensional parameters $\gamma_{q}$, q∈[r], Lindgren and Rue^17^ proposed the use of second order random walk model (RW2) to discretize the Integrated Wiener process prior. Miller et al.^18^ showed that the RW2 model proposed by Lindgren and Rue^17^ can be understood as a penalizing basis expansion of the form $\gamma (u)=\sum_{\;j=1}^{d}\phi_{\;j}(u)\Gamma_{\;j}$ for each γ(⋅) (dropping the subscript q), where the random weights $\Gamma =\{\Gamma_{\;j},j\in [d]\}$ are parameters to be inferred and $\phi_{\;j}(\cdot ),j\in [d]$ are fixed, known basis functions which must be chosen. Yue et al.^19^ note that a similar discretization technique to the one used by Lindgren and Rue^17^ yields the B-spline smoothing with integrated derivative penalty of general order proposed by OSullivan,^20^ and Wood^21^ provide an explicit construction of the corresponding precision matrix. In the method of Lindgren and Rue,^17^ the basis function $\phi_{\;j}(\cdot )$ is chosen to be the linear B-spline function, and the random weights are given zero-mean Gaussian prior with a banded precision matrix $\Sigma^{-1}(\sigma )$ controlled by a single variance parameter σ. In the proposed approach, we use cubic B-splines for the $\phi_{\;j}(\cdot )$ and choose the precision matrix $\Sigma^{-1}(\sigma )$ that is obtained by using an integrated second derivative penalty of Wood.^21^

We put a sum-to-zero constraint such that $\sum_{i=1}^{n}\gamma \left(u_{i}\right)=0$ in all the following example as default. This sum-to-zero constraint is needed since (a) the overall intercept parameter cannot be identified in partial likelihood and (b) the semi-parametric model we introduced above is invariant to addition of any constant. The constraint will not impact the shape of the nonlinear effect estimate, but only shift its location, which gives it an interpretation as an effect relative to the overall level. This linear constraint will be handled using the approach of conditioning by Kriging,^22^ by simulating from the unconditional distributions and applying the corrections to the unconditional samples.

Finally, define the variance parameter vector $\theta =(\theta_{0},\ldots ,\theta_{r})$ where $\theta_{q}=-2\log\;\sigma_{q}$, q=1,…,r, and $\theta_{0}=-2\log\;\sigma_{\xi }$. The variance parameters are given prior distribution θ∼π(θ).

The proposed method can also accommodate the inference of spatial variations, by replacing the covariance function of $\gamma_{q}(u)$ with a proper spatial covariance function. In the following example, we will take the Matern covariance function $M_{\nu }(\|.\|;\sigma_{q},\rho_{q})$ as the default choice, such that $\mathrm{Cov}(\gamma_{q}(u_{i}+h),\gamma_{q}(u_{i}))=M_{\nu }(\|h\|;\sigma_{q},\rho_{q})$ for any $u_{i},h\in R^{2}$. For the Matern covariance function $M_{\nu }(\|.\|;\sigma_{q},\rho_{q})$, we take the same parametrization as in Brown et al.,^23^ with fixed shape parameter ν=1, and $\sigma_{q}$ and $\rho_{q}$, respectively represent the marginal standard deviation parameter of $\gamma_{q}$ and its practical correlation range. The corresponding variance parameter $\theta_{q}$ is defined as $\theta_{q}=(\log\;(\sigma_{q}),\log\;(\rho_{q}))$ in this case.

### 2.3. Partial likelihood

Our inference is carried out via a partial likelihood function. Define the *risk set* $R_{ij}=\{k,l\;:\;y_{kl}\geq y_{ij}\}$, which contains all the alive and uncensored observations across all the groups at time $y_{ij}$. Assuming $y_{ij}\neq y_{kl}$ when (i,j)≠(k,l), the partial likelihood can be written as follows:

(2) $$\begin{array}{rl} \pi (y|\eta ) & =\prod_{i=1}^{n}\prod_{\;j=1}^{n_{i}}\{\frac{\exp\;[\eta_{ij}]}{\sum_{l,k\in R_{ij}}^{}\exp\;[\eta_{lk}]}{\}}^{d_{ij}},\\ & =\prod_{i=1}^{n}\prod_{\;j=1}^{n_{i}}\{\frac{1}{1+\sum_{l,k\in R_{ij},(l,k)\neq (i,j)}\exp\;[\Delta_{lk,ij}]}{\}}^{d_{ij}}, \end{array}$$

where $\Delta_{lk,ij}=\eta_{lk}-\eta_{ij}$. Note that $h_{0}(t)$ does not appear in the partial likelihood, and hence inference may be carried out in the absence of assumptions about $h_{0}(t)$.

The partial likelihood (2) can be written in the following form:

(3) $$\begin{array}{cc} \pi (y|\eta ) & =\prod_{i=1}^{n}\prod_{\;j=1}^{n_{i}}\pi (y_{ij}|\eta ), \end{array}$$

while in order for a model to be compatible with INLA, its likelihood must have the form:

(4) $$\begin{array}{cc} \pi (y|\eta ) & =\prod_{i=1}^{n}\prod_{\;j=1}^{n_{i}}\pi (y_{ij}|\eta_{ij}). \end{array}$$

Stringer et al.^11^ extend this to permit partial likelihoods of the form:

(5) $$\begin{array}{cc} \pi (y|\eta ) & =\prod_{i=1}^{n}\prod_{\;j=1}^{n_{i}}\pi (y_{ij}|\eta_{i}), \end{array}$$

with $\eta_{i}\;:\;=\{\eta_{ij},j\in [n_{i}]\}$, which still does not include (2). Martino et al.^5^ are able to write the likelihood for their Cox PH model in the form (4) using the full, not partial likelihood (2). Because of this, they require assumptions to be made about the baseline hazard.

Further define $\Delta_{lk,ij}=\eta_{lk}-\eta_{ij}$ in terms of the additive predictors (1). Note that $\Delta_{lk,ij}=\Delta_{11,ij}-\Delta_{11,lk}$ for every (i,j,l,k). To simplify notation, define $\Delta_{ij}=\Delta_{11,ij}$, and note that $\Delta_{11}=0$. The entire partial likelihood (2) depends on η only through $\Delta =\{\Delta_{ij}\;:\;i\in [n];j\in [n_{i}]\}$. For the remainder of the paper we reflect this in our notation, writing π(y|Δ)≡π(y|η) and defining the log-likelihood ℓ(Δ;y)=logπ(y|Δ).

In existing Laplace approximations for posterior distributions,^2,5,11^ the *latent parameters* W are defined as W=(Δ,Γ,β,ξ), where the (differenced) linear predictors Δ are included as part of the latent parameter vector. Approximate Bayesian inference of this type requires the precision matrix of W to be non-singular,^24^ and hence a small noise term $\epsilon_{ij}\overset{iid}{∼}\text{N}(0,\tau^{-1})$ (for some large, fixed τ) is added into the model to make the required matrices non-singular. Define the noised linear predictors as

$$\;{\tilde{\Delta }}_{ij}=\eta_{11}-\eta_{ij}+\epsilon_{ij},$$

then the resulting precision matrix of (noised) latent parameters $\tilde{W}\;:\;=(\tilde{\Delta },\Gamma ,\beta ,\xi )$ is non-singular even when improper prior such as the RW2 prior is used.

Such posterior approximation methods have the advantage that, when the likelihood can be factored out in the form of (4), the resulting log-likelihood Hessian matrix is diagonal and hence efficient to be computed and stored.^2^ Alternatively, if the likelihood is in the form of (5), the Hessian matrix is still sparse even it is no longer diagonal.^11^ However, if one considers applying such approximate Bayesian inference on Cox PH model with partial likelihood, the resulting Hessian matrix will be completely dense and with the number of elements growing quadratically with sample size N. The novel inference method that we introduced in this paper on the other hand does not use the noised predictor, and hence will have a Hessian matrix with a fixed dimension that will not grow with the sample size.

## 3. Methods

### 3.1. Approximate Bayesian inference

To deal with the problem of dense Hessian matrix, we proposed a new way to utilize the Laplace approximation, by defining the latent parameter vector to only include the parameters of interest, W=(Γ,β,ξ). As before, the differenced additive linear predictors Δ can be computed from W, but Δ will not be included as part of W. Note that in our definition, the dimension of this latent parameter vector will be constant, and hence the size of the dense Hessian matrix will be small regardless of the sample size N. This will significantly reduce the memory requirement and computational challenge introduced by computing, storing and factorizing the Hessian matrix, which is necessary for the inferential procedures.

When nonlinear semi-parametric covariate effect is included in the model, W will have a singular precision matrix as the precision matrix of Γ is rank deficient, and hence direct application of the Laplace approximation of Tierney and Kadane^24^ will be problematic. This problem of the singular precision matrix is typically fixed by introducing a small Gaussian noise into the additive linear predictors, which makes the precision matrix full rank.^11,2^ In our proposed approach, no noises will be added into the linear predictors; instead we will follow the approach of Wood,^25^ to fix this problem by adding a very small constant term into the diagonal terms of the precision matrix of Γ, $\Sigma_{\Gamma }^{-1}$, which will also result in a full rank precision matrix for W. The proposed modification only shifts the diagonal terms of $\Sigma_{\Gamma }^{-1}$ by a very small constant, hence will not change any conditional independence structure in the original prior.

Define $W|\theta ∼\text{N}[0,Q_{\theta }^{-1}]$, where $Q_{\theta }$ is the precision matrix for W. We are interested in estimating and sampling from the joint posterior distribution of the latent parameters:

(6) $$\begin{array}{c} \pi (W|y)=\int \pi (W|y,\theta )\pi (\theta |y)d\theta . \end{array}$$

We are also interested in the joint posterior distributions of the variance parameters:

(7) $$\begin{array}{c} \pi (\theta |y)=\frac{\int \pi (W,y,\theta )dW}{\int_{}\int_{}\pi (W,y,\theta )dWd\theta }. \end{array}$$

These are used for point estimates and uncertainty quantification of the variance parameter θ, and appear as integration weights in (6).

For the posterior of variance parameter (7), we follow the procedure of Stringer et al.^11^ to approximate it with its corresponding Laplace approximation ${\tilde{\pi }}_{LA}(\theta |y)$. The posterior of the latent parameter vector (6) is approximated by $\tilde{\pi }(W|y)$ defined as:

(8) $$\begin{array}{cc} \tilde{\pi }(W|y) & =\sum_{k=1}^{K}{\tilde{\pi }}_{G}(W|y,\theta^{k}){\tilde{\pi }}_{LA}(\theta^{k}|y)\delta_{k}, \end{array}$$

where $\{\theta^{k},\delta_{k}{\}}_{k=1}^{K}$ is a set of nodes and weights corresponding to an adaptive Gauss-Hermite quadrature rule. The ${\tilde{\pi }}_{G}(W|y,\theta^{k})$ is a Gaussian approximation for $\pi (W|y,\theta^{k})$ and the ${\tilde{\pi }}_{LA}(\theta^{k}|y)$ is a Laplace approximation for $\pi (\theta^{k}|y)$, which we describe at below.

For any fixed θ, define

(9) $$\begin{array}{rl} {\hat{W}}_{\theta }=\left({\hat{\Gamma }}_{\theta },\hat{\beta },{\hat{\xi }}_{\theta }\right) & ={\text{argmax}}_{W}\log\;\pi (W|\theta ,y),\\ H_{\theta }(W) & =-\frac{\partial^{2}}{\partial W\partial W^{T}}\log\;\pi (W|\theta ,y). \end{array}$$

For the conditional posterior

(10) $$\begin{array}{c} \pi (W|\theta ,y)\propto \exp\;\left\{-\frac{1}{2}W^{T}Q_{\theta }W+\ell \left(\Delta ;y\right)\right\}, \end{array}$$

a second-order Taylor expansion of logπ(W|θ,y) about $W={\hat{W}}_{\theta }$ yields a Gaussian approximation:

(11) $$\begin{array}{cc} \pi (W|\theta ,y) & \approx {\tilde{\pi }}_{G}(W|y,\theta )\\ & \propto \;\text{exp}\left\{-\frac{1}{2}{\left(W-{\hat{W}}_{\theta }\right)}^{T}H_{\theta }\left({\hat{W}}_{\theta }\right)\left(W-{\hat{W}}_{\theta }\right)\right\}. \end{array}$$

Define ${\hat{\Delta }}_{\theta }$ be the differenced additive linear predictors computed at ${\hat{W}}_{\theta }$. For the joint posterior of the variance parameters, the method of Tierney and Kadane^24^ yields a Laplace approximation:

(12) $$\pi (\theta |y)\approx {\tilde{\pi }}_{LA}(\theta |y)\propto \pi (\theta )\{\frac{\left|Q_{\theta }\right|}{\left|H_{\theta }({\hat{W}}_{\theta })\right|}{\}}^{1/2}\exp\;\{-\frac{1}{2}{{\hat{W}}_{\theta }}^{T}Q_{\theta }{\hat{W}}_{\theta }+\ell ({\hat{\Delta }}_{\theta };y)\}.$$

With these approximations available, inference for θ can be directly obtained using the analytical form of ${\tilde{\pi }}_{LA}(\theta |y)$ as in equation (12). Inference for W or its marginal component can be easily obtained if it is possible to get independent samples from $\tilde{\pi }(W|y)$.

To sample from $\tilde{\pi }(W|y)$, note that by equation(6), $\tilde{\pi }(W|y)$ is Gaussian mixture distribution with K mixture components each with mixture weight being

$$\phi_{k}={\tilde{\pi }}_{LA}(\theta^{k}|y)\delta_{k},\;k\in [K].$$

For a large integer B, we sample independent $\{Z_{i}{\}}_{i=1}^{B}$ from $\text{Multinomial}(\phi_{1},\ldots ,\phi_{K})$, and then for each $Z_{i}\in [K]$, sample $W_{i}$ from ${\tilde{\pi }}_{G}(W|y,\theta^{Z_{i}})$. The resulting sample $\{W_{i}{\}}_{i=1}^{B}$ then contains B independent draws from $\tilde{\pi }(W|y)$, and all the posterior summaries for W can be obtained using this independent sample.

### 3.2. Adaptive quadrature and automatic differentiation

Computing the approximations (8) requires choosing a quadrature rule consisting of nodes $\{\theta^{k}{\}}_{k=1}^{K}$ and weights $\{\delta_{k}{\}}_{k=1}^{K}$ for some chosen K∈N. Stringer et al.^11^ lay a user-chosen grid over a range of θ that is thought to be plausible, and then compute the Gaussian (11) and Laplace (12) approximations at each point on this grid. This requires the user to choose the location and spread of the grid points, as well as a number K of points that is large enough such that the structure of the resulting posterior approximations is captured. The function π(W|y,θ) must be optimized, and the Hessian matrix must be stored, for each of these K points. In addition to this strategy requiring the user to have specialist knowledge to implement, it is potentially computationally wasteful since K has to be chosen large enough such that the quadrature points densely cover the range where the majority of mass in π(θ|y) lies. In our case, this problem is made more severe by the presence of a dense Hessian. Martino et al.^5^ use the INLA software and its adaptive quadrature rule which avoids the need for the user to choose points but may still result in a large number of points being used for this same reason.

To mitigate the computational challenges associated with applying a manual quadrature rule for (8), we implement Adaptive Gauss-Hermite Quadrature (AGHQ). This technique has been motivated as a useful tool for Bayesian inference^26^ and work has been done to show that it is very accurate when using only a very small number of quadrature points,^28,27^ for example attaining $O(N^{-1})$ asymptotic accuracy with K=3 and $O(N^{-2})$ with K=5. The use of a small number of quadrature points means only a small number of dense Hessian matrices need to be stored in memory, an improvement over Stringer et al.^11^ that is necessary to extend their method to work with the partial likelihood of the Cox PH model.

Computing the AGHQ rule requires computation of the mode of the Laplace approximation:

(13) $$\begin{array}{cc} \hat{\theta } & =\text{argmax}\log\;{\tilde{\pi }}_{LA}(\theta |y), \end{array}$$

as well as the Hessian matrix of $\log\;{\tilde{\pi }}_{LA}(\theta |y)$ and its Cholesky decomposition. For the optimization, we use the TMB package, which implements automatic computation of the Laplace approximation and its gradient, which avoids repeated inner optimizations to find ${\hat{W}}_{\theta }$ at different values of θ.^29^ Because of the use of automatic differentiation algorithm, computing the AGHQ rule in the proposed method will not introduce significant computational difficulty.

## 4. Examples

In this section, we present two simulation studies and two data analysis examples. All the codes are available in the online supplementary materials.

### 4.1. Simulation studies

We will provide two simulation studies to demonstrate the accuracy of our proposed method and under which situations the accuracy is improved over the existing full likelihood method INLA. Also, we will show that the proposed method provides posterior approximations that are comparable to results of MCMC, with much shorter runtime.

#### 4.1.1. Simulation with sparse frailties

**Figure 1.**
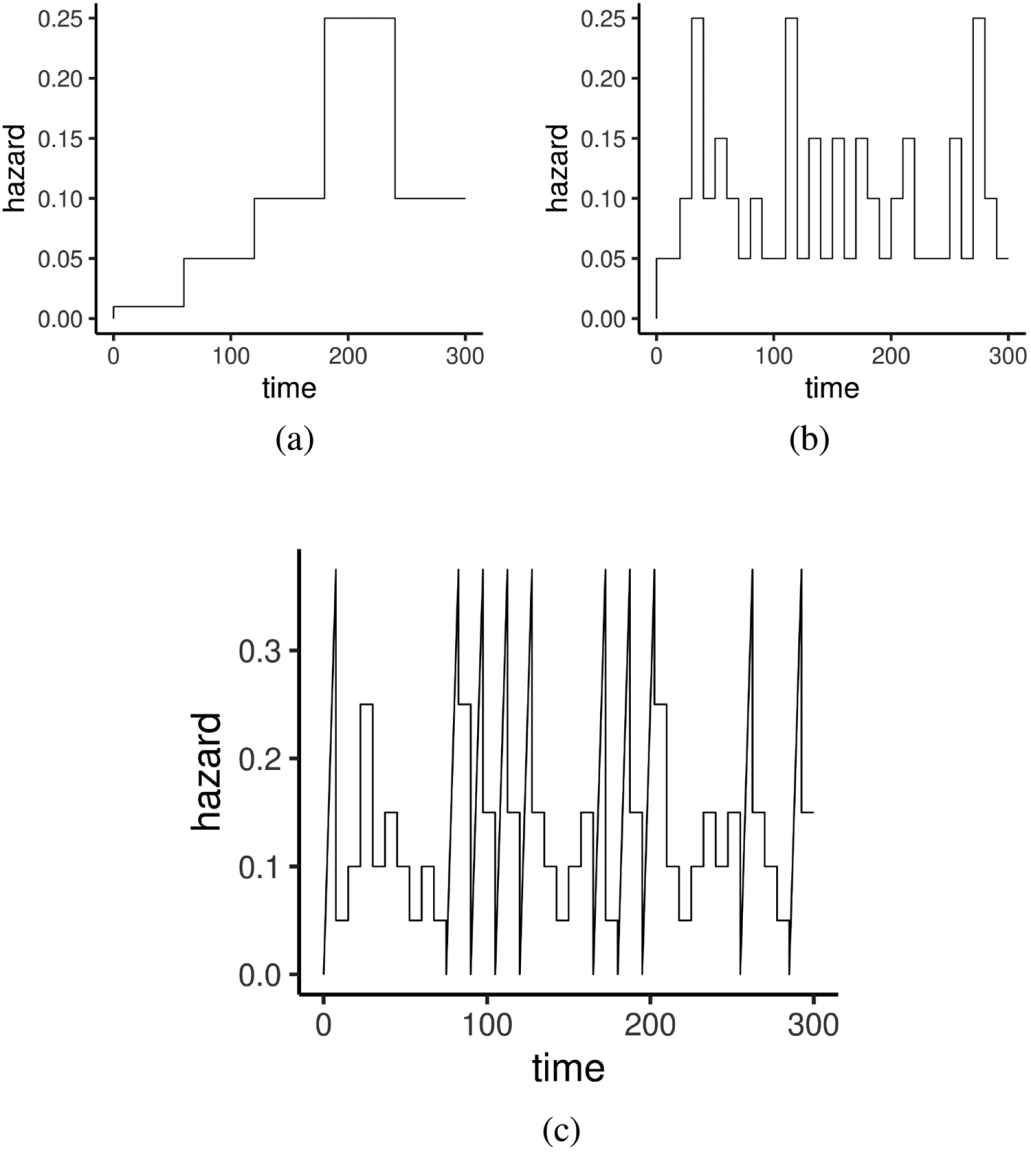
True Baseline Hazards in the two examples in section 4.1. (a) Simple stepwise baseline. (b) Oscillating stepwise baseline. (c) Complicated baseline.

In the first simulation study, we considered the Bayesian inference problem for models with sparse frailties. In other words, survival times were correlated within groups while the number of observations in each group is small. We randomly generated n=60 groups, each group with $n_{i}\equiv m$ observations. The group-level frailties $\left\{\xi_{i},i\in [n]\right\}$ were simulated independently from $N(0,\sigma_{\xi }^{2})$, with varying levels of $\sigma_{\xi }$. Besides the independent frailties, we also assumed there is a covariate x generated from N(0,1), with covariate effect β=0.2. Among all the survival times generated in this study, 10% of observations were randomly selected to be right-censored as default. This corresponds to the random censoring mechanism, which directly implies the assumption of independent censoring holds in the simulation.^13^ In this simulation study, we consider the baseline function to be a simple step function. This choice of piece-wise constant baseline function corresponds to the piece-wise exponential model, which is a type of Cox PH model that is frequently used in the literature.^30^ The baseline hazard function in this simulation study is shown in Figure 1(a). We consider six different levels of frailty sparsity in this simulation study by respectively setting m to 1, 2, 3, 4, 5 and 10. The parameters of primary interest in this study are the group-level frailties.

The fixed effect β was given a prior N(0,1000). The variance parameter $\sigma_{\xi }$ was given an Exponential prior with median of 1, which is a *penalized complexity* prior of Simpson et al.^31^ The same priors were used for implementations of both our proposed method and INLA. For the adaptive quadrature we used in our inference, the number of grid points for variance parameter was set to be K=15. For the implementation of INLA, we used its first-order random walk model for the baseline hazard run under its default settings. To compare the accuracies between the two methods, we used the metrics of posterior mean square error (MSE) and coverage rates of the 95% posterior credible intervals, for both the fixed effect parameter and the frailties. All the metrics were computed by averaging through 5000 independent replications.

**Figure 2.**
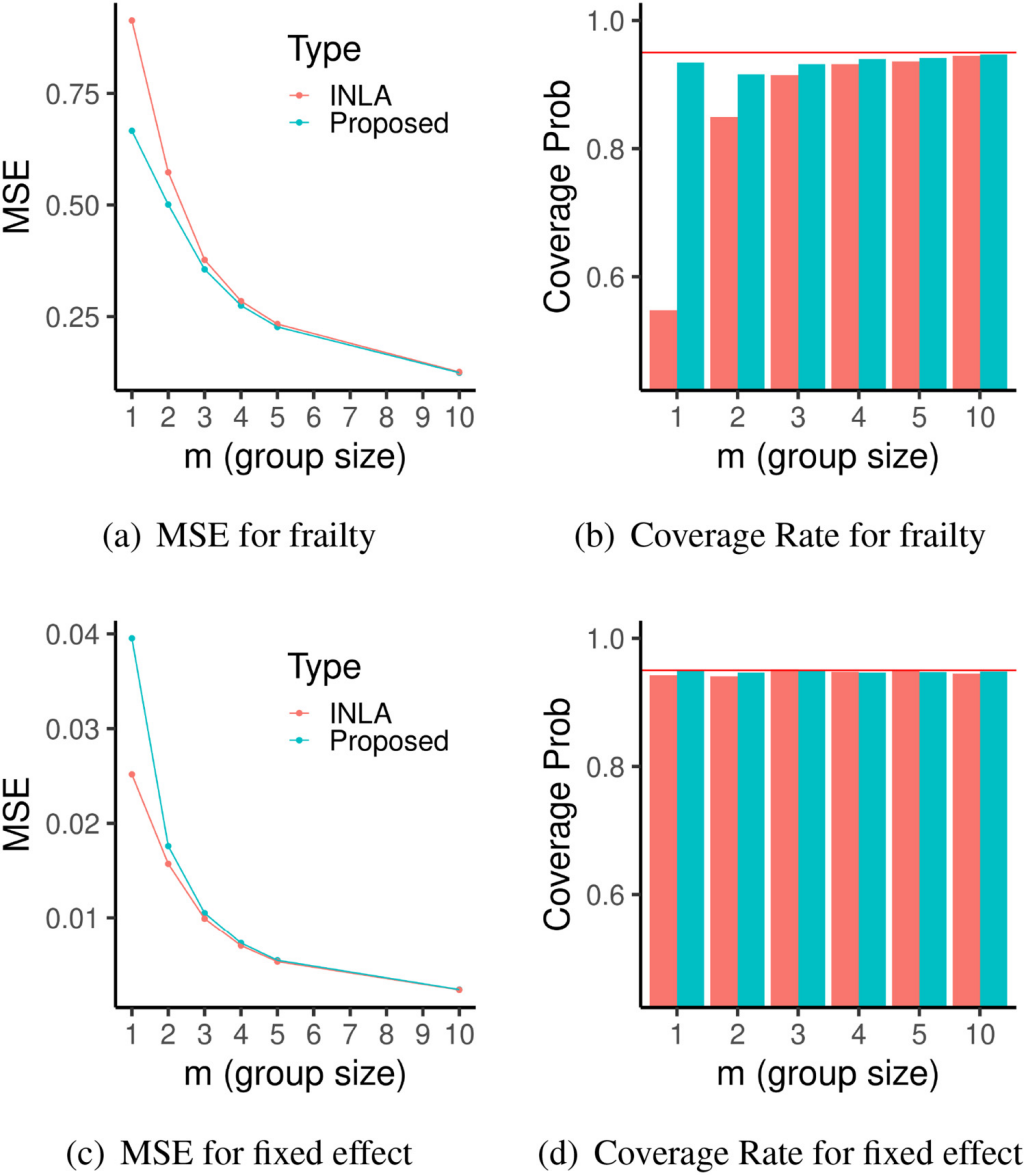
Results for the first simulation with sparse frailty in section 4.1. Left: Plots of MSE of frailties (a) and fixed effect (c) from 5000 independent replications with different m settings, using INLA (red) and the proposed method (blue). Right: Bar-plots of Coverage Rate of 95% posterior credible intervals of frailties (b) and of fixed effect (d) from 5000 independent replications with different m settings, using INLA (red) and the proposed method (blue). The red horizontal line is the nominal rate of 95%. INLA: integrated nested laplace approximation; MSE: mean square error.

The comparison metrics when $\sigma_{\xi }=1$ are shown in Figure (2). Based on Figure (2)(a) and (b), it can be noticed that our proposed method in general gives more accurate inferential results than INLA for frailty effects, both in terms of smaller MSE and coverage rates closer to the nominal level (i.e. 95%), and these differences get larger as the frailties get sparser (smaller m). The comparison metrics for fixed effect are similar between the two approaches, as shown by Figure (2)(c) and (d). When m=1, INLA has slightly better MSE for the fixed effect parameter β than the proposed method, because of the additional correction to the marginal posterior of β implemented in its software.^2^

For completeness, we implemented the same simulation setting for $\sigma_{\xi }=0.4,0.8$, and 1.3. As $\sigma_{\xi }$ grows, we found that as a contrast to the other method, inferences from the proposed method are more robust to the sparse frailties, especially in terms of coverage probability for ξ. The detailed comparison metrics for each setting of $\sigma_{\xi }$ can be found in Supplemental Appendix B. These results show that unless the group level frailties have trivial variation, the proposed method yields more reliable result than INLA, especially when frailties are sparse.

We then study the performance of the two inference methods when the group sizes $n_{i}$’s are varying across groups instead being fixed at m for all groups. Again the results suggest that as long as the majority of the groups have small group sizes, the proposed approach based on partial likelihood will still be more advantageous in the inference of frailties. The detailed results can be found in Supplemental Appendix A.

To compare the performance at different levels of censoring rates, we use the setting of $\sigma_{\xi }=1$ and m=2 at censoring rates of 10%, 20% and 40%, and compare the proposed method with INLA as above. The detailed comparison results can be found in Supplemental Appendix C, which shows that the above conclusion still holds for sparse frailty across different levels of censoring rates. As the censoring rates get higher, both methods have worse results for the inference of ξ measured by MSE and coverage rates, but INLA’s full-likelihood approach is more severely affected.

The simulation result above seems to be related to the type of full likelihood INLA utilized in its inference.^5^ In Cox,^32^ Cox pointed out one problem associated with the use of the type of full likelihood considered by Martino et al.,^5^ that is the large number of parameters introduced in order to model the unknown baseline hazard function. In this simulation study, the sample size is only 60m, but the latent parameter contains more than 60 parameters not counting the additional parameters INLA introduced for modeling the baseline hazard, which is likely to become a problem if m is small.

To demonstrate the accuracy of our proposed approximation and the computational advantage compared to existing method, we also fitted the same partial likelihood model using MCMC method, through STAN’s No U-turn Sampler (NUTS),^33^ using two replications where $\sigma_{\xi }=1$ and m=10 and 2, respectively. With four chains each with 35,000 total iterations with 25,000 warmups running in parallel, MCMC took a total time of 40.89 minutes when m=10 and 3.56 minutes when m=2. As a comparison, the proposed method only took 29.64 seconds when m=10 and 1.31 seconds when m=2, to obtain 10,000 independent samples of W from the approximate posterior. On average, our method can be run in the time that takes to perform 423 iterations of MCMC when m=10, and 215 iterations when m=2.

The difference between posterior distributions yielded by the MCMC method and the approximate posterior obtained by the proposed method is quantified using Kolmogorv–Smirov (KS) statistic, which denotes the maximal absolute difference between the two cumulative posterior distributions, with a larger value indicating less similarity between two distributions. The KS statistics have been computed for both $\sigma_{\xi }$ and β, as well as the mean and maximal KS statistic for the 60 frailties. These results are summarized in Table (1). As illustrated in the table, the proposed method yielded approximate posteriors that are very similar to those yielded by MCMC method, but took a significantly shorter time for computation than MCMC. As m decreases from 10 to 2, the KS statistic for $\sigma_{\xi }$ increases by 0.088. That is because of the use of Laplace approximation for $\pi (\sigma_{\xi }|y)$, which has been known to be less accurate when random effects are sparse.^34^ However, the result from our proposed method is still more accurate than the existing Laplace approximation-based method as shown in Figure (2).

**Table 1.** KS statistic for each parameter in the first simulation study with sparse frailty in the “Examples section”, to compare the proposed approach with MCMC.

Parameters:	$\sigma_{\xi }$	β	$\xi_{i}$
Number of Measurements	KS	KS	max KS	mean KS
m = 2	0.104	0.016	0.059	0.039
m = 10	0.016	0.010	0.033	0.019

KS: Kolmogorv-Smirov; MCMC: Markov Chain Monte Carlo.

#### 4.1.2. Simulation with non-smooth baseline

To compare the accuracy of our method with INLA when the smoothness assumption for baseline hazard function is violated, we performed our second simulation study. We generated n=1000 uncorrelated data points from a distribution with known hazard function. For the baseline hazard functions, we consider three different settings corresponding to three different levels of wiggliness. Specifically, baseline hazard function is respectively set to simple step function, oscillating step function and an extremely complicated function that switches between linear and constant. Again, the first two piece-wise constant baseline hazards correspond to piecewise Exponential models with different complexity.^30^ All of the three baseline hazards $h_{0}(t)$ are shown in Figure (1). The additive predictor is $\eta_{i}=\gamma (u_{i})$ with γ(u)=1.5[sin(0.8u)+1] in all the three simulation settings. We generated the covariates u as $u_{i}\;\overset{ind}{∼}\;\text{Unif}(-6,6)$, i∈[n] and randomly censored 10% of all the survival times.

To infer the unknown risk function γ, we used the Bayesian cubic B-spline smoothing method mentioned in the section 2 in our proposed method, with 50 equally spaced knots. For the smoothing method in INLA, we placed the values of u into 50 discrete bins, and fitted its second-order random walk model for γ.^17^ As shown in Stringer et al.,^11^ the Bayesian semi-parametric smoothing methods we considered here are not sensitive to the choice of number and placement of knots. As before, we implemented INLA under its default setting, with a first-order random walk model for the baseline hazard. This implicitly assumes that $h_{0}(t)$ is smooth. In contrast, our procedure does not infer $h_{0}(t)$, and does not make assumptions about its smoothness. In both of the smoothing methods, the single variance parameter σ that controls the smoothness of γ, was modeled with an Exponential( λ) prior with λ chosen such that P(σ>2)=0.5. For the adaptive quadrature we used in our inference, the number of grid points for variance parameter to is set to be K=7. As in the first simulation study, we compared the accuracy of our proposed method with INLA, using the metrics of MSE computed using posterior mean and coverage rate computed using the 95% posterior credible interval. These metrics were computed from 1000 independent replications.

**Figure 3.**
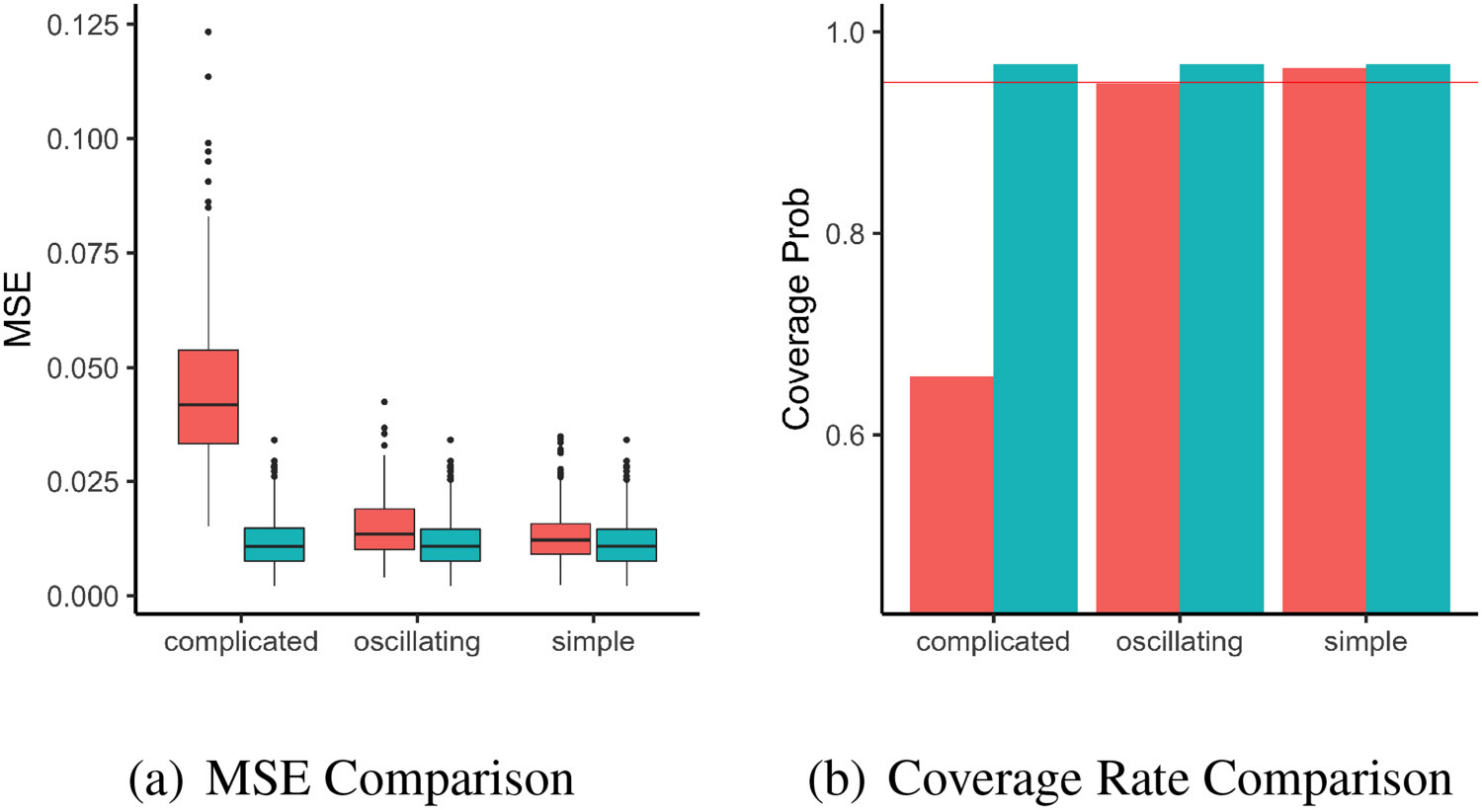
Results for the second simulation with non-smooth baseline in the section 4.1.2. (a): Plots of MSE from 1000 replications with different baseline settings, using INLA (red) and the proposed method (blue). (b): Bar-plot of Coverage Rate of 95% posterior credible intervals from 1000 replications with different baseline settings, using INLA (red) and the proposed method (blue). The red horizontal line is the nominal rate of 95%. INLA: integrated nested laplace approximation; MSE: mean square error.

The comparison metrics under the three settings of baseline hazard are shown in Figure (3). Figure (3)(a) shows boxplots of the mean squared errors provided by the two methods for $\{\gamma (u_{i}){\}}_{i=1}^{n}$ in each replication, for the three different settings of baseline hazard function. Based on the boxplots, the proposed method provides inference results that are at least as accurate as INLA in terms of MSE in all the settings. At the same time, as shown in Figure (3)(b) the proposed method provides consistent coverage rate that is close to the nominal level 95% in all the settings, but INLA has coverage rate lower than the nominal level when baseline hazard is complicated.

While the performance of the proposed method is not affected by the choice of true baseline hazard function, the performance of INLA is sensitive to the true baseline hazard function, as shown in the corresponding boxplots and barplots. This is not unexpected as the full-likelihood used in INLA’s inference implicitly requires that the baseline hazard is smooth enough to be approximated well by its first-order random walk, which will not hold under setting such as Figure (1)(c) where the baseline hazard is varying rapidly as time changes. On the other hand, the inference of our proposed method relies on the partial likelihood, which makes no assumption on the form of the baseline hazard, and hence unaffected by the wiggliness of the baseline hazard in this study.

Again, to assess the accuracy of the proposed posterior approximation, we fitted the same partial likelihood model using MCMC using NUTS with four chains each with 10,000 iterations and 8000 warmups, for an arbitrarily chosen replication. The KS statistic for variance parameter σ that controls the smoothness of the inferred γ is 0.035, and the maximum and mean KS statistics for the risk function evaluations $\{\gamma (u_{i}){\}}_{i=1}^{n}$ are 0.027 and 0.021. We also computed the mean and maximum absolute difference between the posterior mean for $\{\gamma (u_{i}){\}}_{i=1}^{n}$ given by the proposed method and by MCMC, which are respectively 0.002 and 0.007. The small KS statistics and absolute difference between posterior mean demonstrate that the proposed posterior approximation is comparable to MCMC. On the other hand, the average runtime of each Markov chain was around 18.7 hours for the MCMC method to get 10,000 iterations, but the proposed method with 10,000 independent samples only requires 28.3 seconds. On average, our method can be run in the time that takes to perform 8 iterations of MCMC.

### 4.2. Kidney catheter data

Therneau et al.^35^ analyzed a Kidney Catheter dataset using their proposed penalized partial likelihood method. The Kidney Catheter dataset contains 76 times to infection at the point of insertion of a catheter, for n=38 patients. An observation for the survival time of a kidney is censored if the catheter is removed for reasons other than an infection. Each patient i=1,…,n forms a group, and the survival times are the time to infection of each patient’s $n_{i}=2$ kidneys. Because of the small group sizes in the data, this is therefore a practical example of the type of sparse frailty model on which the partial likelihood approach performed better than the full likelihood approach in the simulations of the section 4.1.

We first analyzed this dataset on partial likelihood using the proposed method, with N(0,1000) priors on the linear covariate effects for age, sex and pre-existing disease types. Subject-specific intercepts $\xi_{i}\overset{\overset{iid}{∼}}{N}(0,\sigma_{\xi }^{2})$ were included to account for correlation between kidneys from the same subject. We used an Exponential prior distribution for $\sigma_{\xi }$ with median 2. For the adaptive quadrature we used in our inference, the number of grid points for variance parameter to is set to be 15. Ties are handled using the method of Breslow.^36^ As a comparison, we also used INLA to fit a Cox PH model to these grouped data on its full likelihood, with the same set of priors as above. The setting for baseline hazard is set to default in INLA’s implementation.

**Figure 4.**
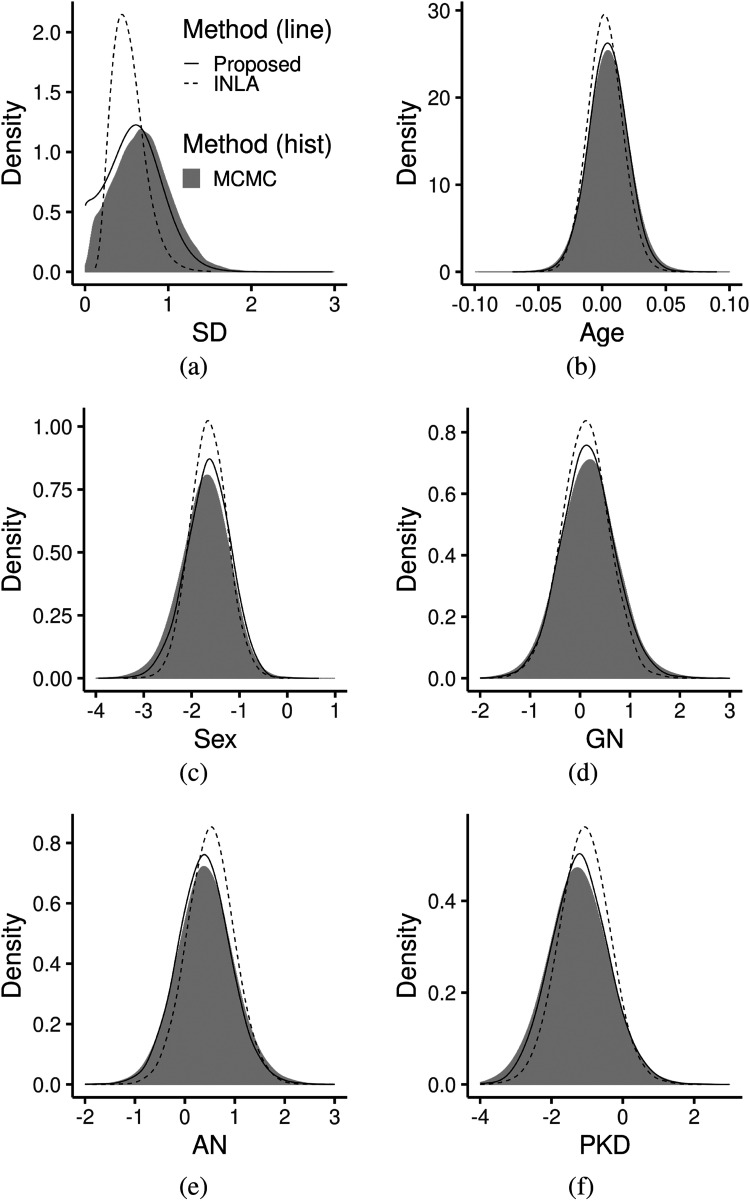
Posteriors of parameters for the kidney data in the section 4.2. Posterior distribution was obtained using the proposed method(solid line), using MCMC(gray histogram) and using INLA (dashed). (a) Posteriors of *σ_ξ_*. (b) Posteriors of *β*_Age_. (c) Posteriors of *β*_Sex_. (d) Posteriors of *β*_GN_. (e) Posteriors of *β*_AN_. (f) Posteriors of *β*_PKD_. MCMC: Markov Chain Monte Carlo; INLA: integrated nested Laplace approximation.

Table (2) shows the results of our procedure compared to the results obtained using INLA and MCMC. Based on the table, our procedure gave different posterior means and reported larger posterior standard deviations compared to INLA, especially for the effects of different disease types. These results are also summarized in Figure (4), where the posteriors obtained from the proposed method are shown to be much closer to MCMC than posteriors obtained from INLA. As we have shown in the section 4.1, when sparse frailties exist, Bayesian inference on partial likelihood tends to be more stable than on full likelihood.

**Table 2.** Estimated means and standard deviations of linear effects by proposed method, INLA and MCMC for the kidney data in the section 4.2.

Methods:		Proposed		INLA		MCMC	
Variables/Reference	Levels	Mean	SD	Mean	SD	Mean	SD
Age		0.00484	0.0147	0.00235	0.0130	0.00516	0.0158
Sex/Male	Female	−1.65	0.473	−1.64	0.385	−1.72	0.507
Disease Type/Other	GN	0.172	0.538	0.111	0.474	0.172	0.576
	AN	0.394	0.542	0.519	0.467	0.415	0.573
	PKD	−1.19	0.826	−1.06	0.708	−1.26	0.859

INLA: integrated nested Laplace approximation; MCMC: Markov Chain Monte Carlo.

Again, to quantify the difference between the proposed posterior approximation and the posterior yielded by MCMC, we computed the KS statistics for the five fixed covariate effects, the 38 patient level frailties and the variance parameter $\sigma_{\xi }$. The KS statistic for $\sigma_{\xi }$ is 0.083, and the mean/maximal KS statistics for fixed effects and frailties are 0.034/0.069 and 0.036/0.051, respectively. As a comparison, when using INLA, the KS statistics for $\sigma_{\xi }$ is 0.357, and the mean/maximal KS statistics for fixed effects and frailties are respectively 0.102/0.127 and 0.143/0.278.

The KS statistics show that the proposed method give similar posteriors to MCMC method, for most of the parameters. As shown by the KS statistics, the posterior for the variance parameter $\sigma_{\xi }$ is approximated less accurately compared to other parameters, because of the problem with Laplace approximation for sparse frailties.^34^ This deviation can also be noticed from Figure (4)(a). However, the inference for $\sigma_{\xi }$ obtained from the proposed method is still closer to MCMC result than that from INLA for all the parameters. The runtimes are respectively 0.64 seconds for our approach with 10,000 independent samples and 1.98 minutes for MCMC with 35,000 iterations with 25,000 warmups. The number of MCMC iterations that can be obtained with the runtime of the proposed method is only 157.

### 4.3. Leukemia data

In this example, we implemented our proposed procedure to fit a Cox PH model to the Leukemia data set analyzed by Martino et al.^5^ as well as previously by Lindgren et al.^37^; Henderson et al.^38^ The dataset contains information from n=1043 adult Leukemia patients, with 16% of observations right-censored. We are interested in quantifying the relationship between survival rate of Leukemia patients with the Townsend deprivation index (tpi), and with their residence locations, controlling effect of the age of the patient, the count of white blood cells at diagnosis (wbc), and sex of the patients.

Based on the model comparison result from Kneib and Fahrmeir,^39^ we follow the same analysis in Martino et al.^5^ to model the effects of age, sex and white blood cell count linearly, and model the effect of the deprivation index (tpi) nonlinearly with the semi-parametric model described in the section 2 using 50 equally spaced knots. Prior distributions $\beta \overset{iid}{∼}N(0,1000)$, were used for the linear regression coefficients. The semi-parametric nonlinear effects $\gamma_{1}(tpi)\;:\;=\{\gamma_{1}({\mathrm{tpi}}_{i}),i\in [n]\}$ were modeled with the reference constraint $\sum_{i=1}^{n}\gamma_{1}({\mathrm{tpi}}_{i})=0$. The single variance parameter $\sigma_{1}$ was given an Exponential prior with a prior median of 2. Furthermore, let M denote the studied region in this dataset, $s\;:\;=\{s_{i},i\in [n]\}\subset M$ denote the residence locations and $\gamma_{2}(s)\;:\;=\{\gamma_{2}(s_{i}),i\in [n]\}$ be their corresponding spatial effects. To model the spatially varying Gaussian process $\gamma_{2}(s)$, a Matern covariance function $M_{\nu }(\|.\|;\sigma_{2},\rho )$ is used. For this Matern covariance function, we follow the procedure of Lindgren et al.^37^ to fix its shape parameter ν at 1, and use independent Exponential priors on the standard deviation parameter $\sigma_{2}$ and correlation parameter ρ such that $P(\sigma_{2}>1)=P(\rho <20\;\text{km})=0.5$. For the adaptive quadrature we used in our inference, the number of grid points is set to be K=4.

**Figure 5.**
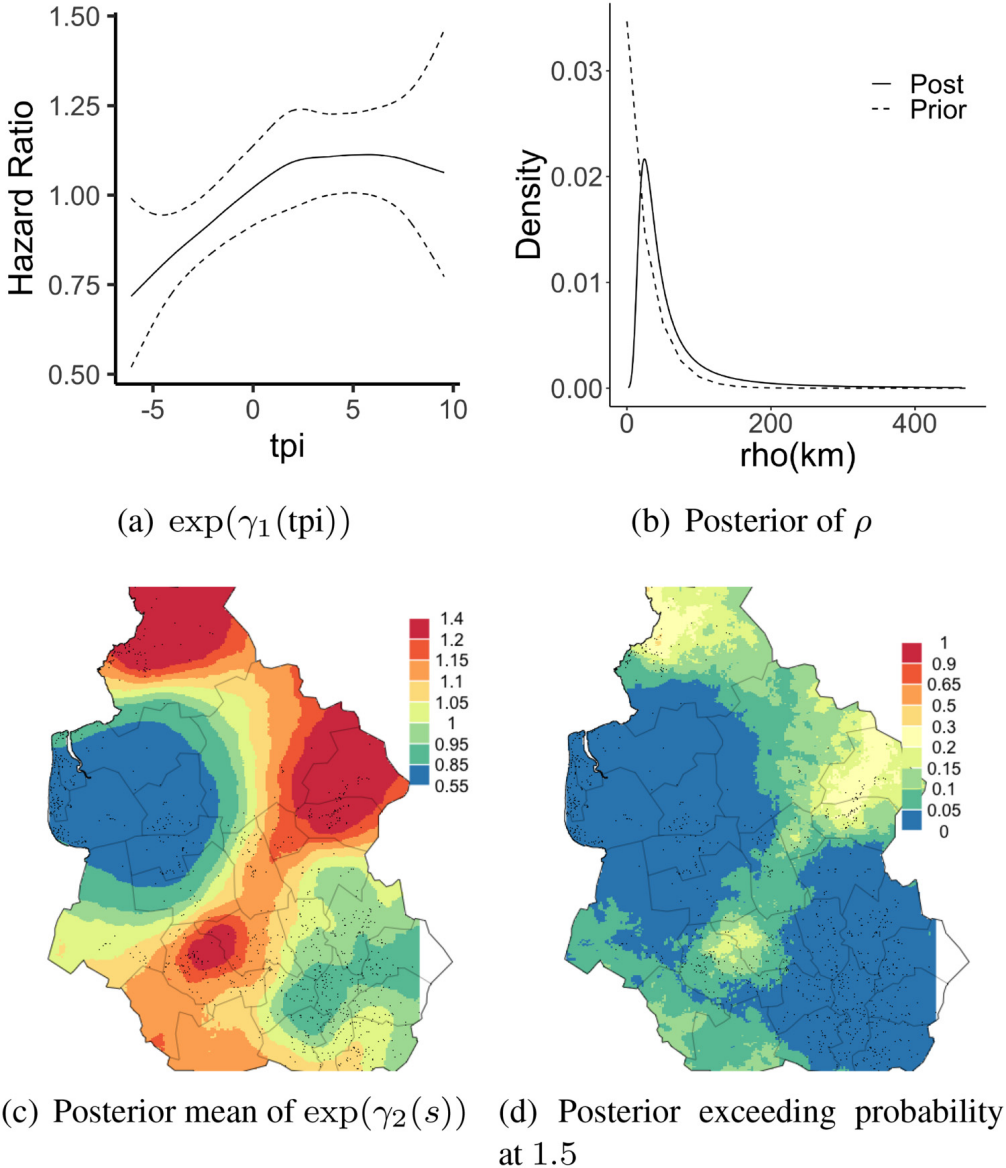
Results for the leukemia data in the section 4.3, (a): Posterior mean of (exponentiated) tpi effect (solid) and its 95% credible interval (dashed). (b) Posterior (solid) and prior (dashed) for the spatial ρ parameter. (c): Posterior mean of (exponentiated) effects of residence locations (d): Posterior probability of the (exponentiated) effect of residence locations being larger than 1.5.

Figure 5(a) shows the posterior mean and posterior 95 % credible interval of the exponentiated covariate effect of tpi using the proposed approach. Figure 5(b) shows the posterior and prior for the spatial correlation parameter ρ in unit of km. To better understand the spatial variation of risk, we simulate $\gamma_{2}(s^{*})\;:\;=\{\gamma_{2}(s_{i}^{*}),i\in [L]\}$ for a large L from its corresponding posterior predictive distribution, with $s_{i}^{*}$ being an arbitrarily chosen point from the region of interest M. This is done by first simulating $\gamma_{2}(s)$ from $\gamma_{2}(s)|y$ using the method described in the section 3, and then simulating $\gamma_{2}(s^{*})$ from $\gamma_{2}(s^{*})|\gamma_{2}(s)$ using the method of Schlather et al.^40^ For each $s_{i}^{*}$, we simulate 100 independent samples, and their corresponding (exponentiated) posterior means and posterior probabilities of having effects exceeding 1.5 are shown in Figure 5(c) and (d). Based on the figure, it can be observed that the exponentiated spatial effect will likely be upper bounded by 1.5 in most of the areas and the isolated areas of high exceeding probability stand out clearly. Posterior exceeding probability at other levels can be found at Supplemental Appendix D.

There are two major differences between our analysis and the analysis in Martino et al.^5^ First, our analysis is carried out on partial likelihood using the proposed method, but the analysis in Martino et al.^5^ is carried out on full likelihood and hence requires assumption on the form of true baseline hazard function. Second, the proposed approach is able to achieve higher resolution estimate for the spatial variation than the approaches taken by Martino et al.^5^ because we include the full continuously sampled locations $\gamma_{2}(s)$ in the latent parameter vector of our analysis without using any approximation to the continuous process. Since $\gamma_{2}(s)$ has a dense precision matrix, the method of Martino et al.^5^ approximates $\gamma_{2}(s)$ with a piece-wise constant function with sparse precision matrix. Therefore, Martino et al.^5^ considers an approximation of the original spatial variation process in order to retain sparseness of the log posterior Hessian, while our model considers the exact Matern process which yields a dense log posterior Hessian, a computational burden that we have already overcome in our procedure.

To contrast the proposed inference method with the method of Stringer et al.,^11^ we compared the runtimes of Cholesky factorization as well as the memory requirements for storage, of $H_{\theta }({\hat{W}}_{\theta })$ obtained by the two methods at a given θ. The $H_{\theta }({\hat{W}}_{\theta })$ obtained by the proposed approach takes 0.02 seconds for a single Cholesky factorization, while the $H_{\theta }({\hat{W}}_{\theta })$ obtained by directly applying the approach of Stringer et al.^11^ takes 1.08 seconds. As for the memory requirements, $H_{\theta }({\hat{W}}_{\theta })$ obtained by the proposed approach requires 13.7 Mb of memory for its storage, while the one obtained by directly applying the approach of Stringer et al.^11^ requires 45.4 Mb to store a single matrix. Since $H_{\theta }({\hat{W}}_{\theta })$ needs to be repeatedly computed and stored during the optimization in equation (9), the computational differences between the two approaches will accumulate during the inference procedures. Therefore, these computational differences demonstrate the necessity of the methodological improvements in our proposed approach as described in the section 3, in order to extend the approach of Stringer et al.^11^ to general Cox PH model, especially for complex datasets as above.

## 5. Discussion

The methodology we proposed in this paper provides a flexible way to carry out Bayesian inference for Cox PH models with partial likelihood, that accommodates the inference for nonlinear covariate effects, spatial variations and correlated survival times. The use of partial likelihood does not require any assumption on the baseline hazard function, which is an advantage over existing approaches for Bayesian inference in this model. We have demonstrated the accuracy and the computational efficiency of our new approach through simulation studies and analysis of two classical datasets in survival analysis. Our proposed method is an appealing option to adopt for the analysis of time-to-event data, when the inference of baseline hazard is not of primary interest.

As shown in our first simulation example, when frailties are sparse, Laplace approximation-based methods tend to yield less accurate approximation for $\pi (\sigma_{\xi }|y)$, a problem that is also pointed out in Ogden.^34^ For such an application, if the inference of the variance parameter $\sigma_{\xi }$ is of primary interest, MCMC type method might be preferred for higher inference accuracies, at the cost of longer runtime. However, we have shown that the proposed approach under such setting yields more accurate inferences than the existing Laplace approximation-based method. Therefore for the analysis with sparse frailties, if the primary interest is the inference of π(W|y) instead of $\pi (\sigma_{\xi }|y)$, or if computational efficiency is of major concern, the proposed method then should be considered as a competent alternative to MCMC method.

The framework of this proposed methodology can be extended to fit more complex models, by modifying the covariance structure of the covariate with nonlinear semi-parametric effect or the covariance structure of the spatial variations. Because we accommodate the dense Hessian matrix of the log-likelihood, our approach could be extended to approximate Bayesian inference for other models with a dense Hessian matrix. We leave such extensions to future work. A R package that implements our proposed method called *abcoxp* is available at https://github.com/AgueroZZ/abcoxpGitHub.

## Supplemental Material

sj-pdf-1-smm-10.1177_09622802221134172 - Supplemental material for Bayesian inference for Cox proportional hazard models with partial likelihoods, nonlinear covariate effects and correlated observations Click here for additional data file. Supplemental material, sj-pdf-1-smm-10.1177_09622802221134172 for Bayesian inference for Cox proportional hazard models with partial likelihoods, nonlinear covariate effects and correlated observations by Ziang Zhang, Alex Stringer, Patrick Brown and Jamie Stafford in Statistical Methods in Medical Research
