# Supplementary material for: Bayesian inference for Cox proportional hazard models with partial likelihoods, nonlinear covariate effects and correlated observations
Source: Stat Methods Med Res. 2022 Nov 1;32(1):165–80. doi: 10.1177/09622802221134172 (PMC9814026; doi:10.1177/09622802221134172)
Supplement: sj-pdf-1-smm-10.1177_09622802221134172 - Supplemental material for Bayesian inference for Cox proportional hazard models with partial likelihoods, nonlinear covariate effects and correlated observations [file sj-pdf-1-smm-10.1177_09622802221134172.pdf]

Supplementary Materials to Bayesian Inference for Cox Proportional Hazard Models with Partial Likelihoods, Nonlinear Covariate Effects and Correlated Observations

Ziang Zhang, Alex Stringer, Patrick Brown, James Stafford

1 Appendix A: Comparison metrics with unequal group sizes for the first simulation example

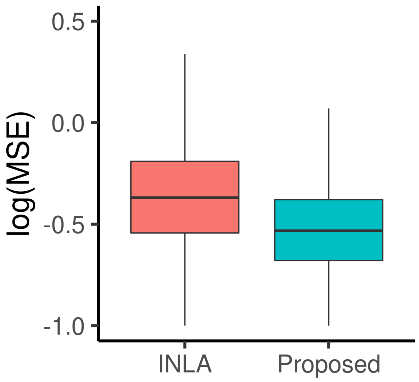

(a) MSE for frailty

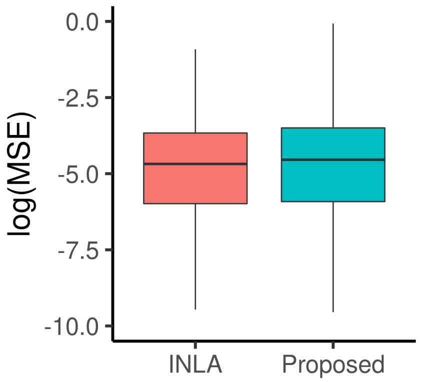

(b) MSE for fixed effect

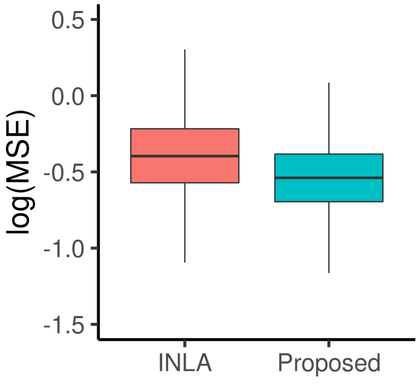

(c) MSE for frailty

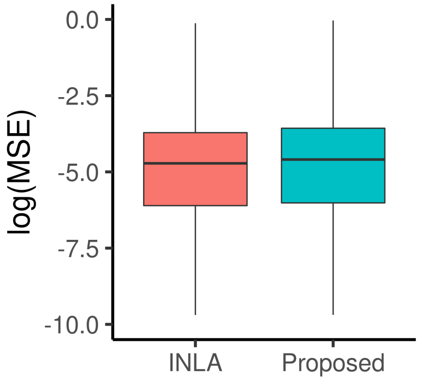

(d) MSE for fixed effect

Figure 1: Results for the first simulation with unequal group sizes. Plots of MSE of frailties and fixed effect from 5000 independent replications using INLA (red) and the proposed method (blue). Upper row: 30 groups with size 1, 25 groups with size 2 and 5 groups wize size 3. Lower row: 30 groups with size 1, 28 groups with size 2 and 2 groups with size 6.

2 Appendix B: Comparison metrics with different σ_ξ for the first simulation example

Number of Measurements	ξ Coverage Rate (Proposed/INLA)	ξ MSE (Proposed/INLA)	β Coverage Rate (Proposed/INLA)	β MSE (Proposed/INLA)
m = 1	0.906/0.478	1.103/1.533	0.950/0.926	0.038/0.028
m = 2	0.918/0.868	0.638/0.744	0.954/0.936	0.018/0.016
m = 3	0.934/0.926	0.423/0.444	0.944/0.944	0.011/0.010
m = 4	0.939/0.934	0.316/0.324	0.944/0.944	0.008/0.008
m = 5	0.941/0.938	0.263/0.266	0.946/0.944	0.006/0.005
m = 10	0.943/0.944	0.144/0.145	0.936/0.938	0.003/0.003

Table 1: Comparison metrics in terms of MSE and posterior coverage rate when $\sigma_\xi = 1.3$, for the 60 frailty effects and the fixed effect in the first simulation study in section 4.1.

Number of Measurements	ξ Coverage Rate (Proposed/INLA)	ξ MSE (Proposed/INLA)	β Coverage Rate (Proposed/INLA)	β MSE (Proposed/INLA)
m = 1	0.952/0.674	0.463/0.569	0.954/0.954	0.043/0.026
m = 2	0.914/0.849	0.384/0.421	0.952/0.948	0.016/0.014
m = 3	0.931/0.910	0.292/0.308	0.950/0.950	0.010/0.009
m = 4	0.935/0.924	0.239/0.248	0.952/0.958	0.007/0.007
m = 5	0.940/0.934	0.227/0.233	0.932/0.938	0.006/0.005
m = 10	0.945/0.943	0.112/0.114	0.952/0.956	0.002/0.002

Table 2: Comparison metrics in terms of MSE and posterior coverage rate when $\sigma_\xi = 0.8$, for the 60 frailty effects and the fixed effect in the first simulation study in section 4.1.

Number of Measurements	ξ Coverage Rate (Proposed/INLA)	ξ MSE (Proposed/INLA)	β Coverage Rate (Proposed/INLA)	β MSE (Proposed/INLA)
m = 1	0.986/0.943	0.252/0.145	0.948/0.962	0.044/0.025
m = 2	0.941/0.909	0.145/0.137	0.954/0.956	0.013/0.012
m = 3	0.930/0.906	0.126/0.125	0.954/0.956	0.008/0.008
m = 4	0.919/0.899	0.116/0.117	0.944/0.956	0.006/0.006
m = 5	0.916/0.901	0.106/0.107	0.928/0.930	0.005/0.005
m = 10	0.940/0.936	0.072/0.073	0.954/0.950	0.002/0.002

Table 3: Comparison metrics in terms of MSE and posterior coverage rate when $\sigma_\xi = 0.4$, for the 60 frailty effects and the fixed effect in the first simulation study in section 4.1.

3 Appendix C: Comparison metrics with different censoring rates for the first simulation example

Censoring Rate	ξ Coverage Rate (Proposed/INLA)	ξ MSE (Proposed/INLA)	β Coverage Rate (Proposed/INLA)	β MSE (Proposed/INLA)
10%	0.916/0.850	0.501/0.573	0.947/0.941	0.018/0.016
20%	0.905/0.813	0.547/0.642	0.941/0.937	0.019/0.017
40%	0.879/0.731	0.655/0.776	0.942/0.934	0.023/0.021

Table 4: Comparison metrics in terms of MSE and posterior coverage rate when $\sigma_\xi = 1$ and $m = 2$, for the 60 frailty effects and the fixed effect in the first simulation study in section 4.1, at censoring rate of 10%, 20% and 40%.

4 Appendix D: Posterior exceeding probability for the Leukemia Data analysis

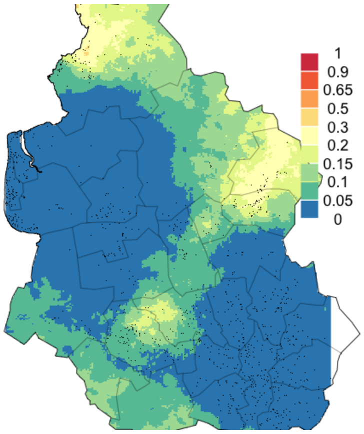

(a) Posterior probability of $\exp(\gamma_2(s_i)) > 1.5$

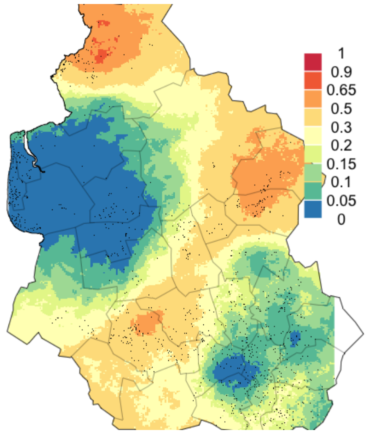

(b) Posterior probability of $\exp(\gamma_2(s_i)) > 1.2$

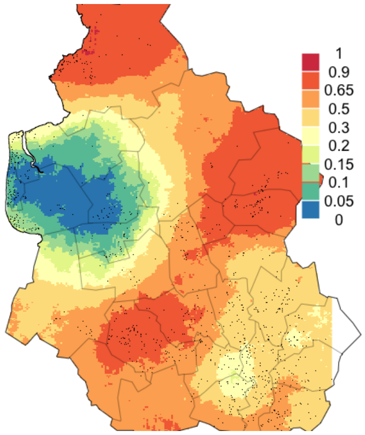

(c) Posterior probability of $\exp(\gamma_2(s_i)) > 1.0$

Figure 2: Additional results for the leukemia data. Posterior probability of the exponentiated effect of residence locations being larger than (a) 1.5, (b) 1.2 and (c) 1.
